# Working Memory Capacity as a Factor Influencing the Relationship between Language Outcome and Rehabilitation in Mandarin-Speaking Preschoolers with Congenital Hearing Impairment

**DOI:** 10.3389/fpsyg.2017.00357

**Published:** 2017-03-09

**Authors:** Ming Lo, Pei-Hua Chen

**Affiliations:** Speech and Hearing Science Research Institute, Children’s Hearing FoundationTaipei, Taiwan

**Keywords:** working memory, hearing impairment, receptive language, expressive language, child development, Mandarin-speaking preschoolers

## Abstract

Memory processes could account for a significant part of the variance in language performances of hearing-impaired children. However, the circumstance in which the performance of hearing-impaired children can be nearly the same as the performance of hearing children remains relatively little studied. Thus, a group of pre-school children with congenital, bilateral hearing loss and a group of pre-school children with normal hearing were invited to participate in this study. In addition, the hearing-impaired participants were divided into two groups according to their working memory span. A language disorder assessment test for Mandarin-speaking preschoolers was used to measure the outcomes of receptive and expressive language of the two groups of children. The results showed that the high-span group performed as good as the hearing group, while the low-span group showed lower accuracy than the hearing group. A linear mixed-effects analysis showed that not only length of rehabilitation but also the memory span affected the measure of language outcome. Furthermore, the rehabilitation length positively correlated with the measure of expressive language only among the participants of the high-span group. The pattern of the results indicates that working memory capacity is one of the factors that could support the children to acquire age-equivalent language skills.

## Introduction

A number of demographic, instrument and medical factors, such as socioeconomic status, parent-children interaction, use of hearing devices and participation in rehabilitation programs, have been identified to relate to language functioning in children with hearing impairment (Geers et al., 2003; Niparko et al., 2010). However, an enormous variability in language performances of hearing-impaired children was not fully explained by the above factors. A body of evidence emerged showing that memory processes could account for a significant part of the unexplained variance (Rönnberg et al., 2008, 2013; Pisoni et al., 2016). For instance, research based on the Ease Language Understanding model has shown that working memory demand in a speech recognition task is higher when the incoming auditory information is degraded or distorted. In other words, the association between working memory capacity and speech recognition becomes stronger for those who are hearing-impaired. From the new point of view, the language deficits in children with hearing loss can be attributed to not only the disorders in sensory system but also the strength of cognitive ability.

For ordinary children, growth in language skills depends partly on working memory ability (Baddeley and Wilson, 1993; Adams and Gathercole, 2000; Archibald and Gathercole, 2006; Montgomery and Windsor, 2007). Children have to rely on their working memory to retain language inputs provided by adults and peers during the course of language development. Children make sense of the inputs by integrating the retained information with the knowledge retrieved from their own long-term memory or with other pieces of information received at the time being. The capacity of working memory has a limit, however. Processing and storage of information share a common pool of resource. Less information can be stored if more processing is in demand, and vice versa (Souza et al., 2015). Influence of the memory ability on language development begins in quite early phase of one’s life. For instance, recognition memory at infancy has been shown to correlate with vocabulary size at 12 and 36 months (Rose et al., 2009). Moreover, this kind of relationship continues throughout not only the pre-school phase but also the school years of one’s development of language proficiency (Fagan and McGrath, 1981; Thompson et al., 1991).

Language skills can be divided into receptive and expressive abilities, and the two types of language skills dissociate to some extent (Clark, 1995). For average children at infancy and in early childhood, receptive language precedes expressive language (Goldin-Meadow et al., 1976; Benedict, 1979; Gershkoff-Stowe and Hahn, 2013) and the size of receptive vocabulary is larger than the size of expressive vocabulary (Fenson et al., 1994). Therefore, receptive and expressive skills were usually measured separately in language assessment of hearing-impaired children (Mayne et al., 1998a,b). However, only a few studies included a sample of hearing peers as the control group to compare with children who were deaf and hard of hearing (Niparko et al., 2010; Yoshinaga-Itano et al., 2010; Vohr et al., 2012; Li et al., 2014). The results were not entirely consistent across studies.

Vohr et al. (2012) assessed language skills of children with mild to profound hearing loss at ages between 4:7 and 5:5, and the results of the assessment showed that the hearing-impaired children had lower scores of receptive and expressive language skills than hearing children. The same pattern of the group differences was observed by Niparko et al. (2010). The children in that study received cochlear implantation before 5 years old. Their receptive and expressive skills were evaluated 3 years after the implantation, and the mean scores of both measures were lower than age-appropriate hearing peers. A similar experimental design was employed by Li et al. (2014) to measure receptive and expressive vocabulary of children who received cochlear implantation at ages between 2 and 3 years old. The assessment at age of 4:6 showed that the hearing-impaired children were not on a par with their hearing peers. In contrast to the above findings, Yoshinaga-Itano et al. (2010) obtained a different pattern of group differences from children with severe or profound hearing loss. The researchers found that the children’s receptive language skill at 7 years old was nearly the same as children with normal hearing, although there was a small delay in expressive vocabulary. Among the above four studies, only Yoshinaga-Itano et al. (2010) provided information concerning cognitive ability of their participants. According to the description of participant characteristics, the cognitive ability of more than 90% of the participants was in the normal range. However, details of what aspect of cognitive ability has been measured were not depicted.

A set of previous studies have shown that children with congenital hearing loss have lower scores on the tests of working memory than do children with normal hearing (Pisoni and Cleary, 2003; Pisoni et al., 2011). Moreover, another line of research has revealed that language deficits in hearing-impaired children could be related to low capacity of working memory (Pisoni and Geers, 2000; Dawson et al., 2002; Kronenberger et al., 2013). It was possible that the language performance observed by Yoshinaga-Itano et al. (2010) could be attributed to the children’s working memory capacity. That is, the participants had sufficient working memory capacity to support the participants to acquire age-equivalent language skills. This hypothesis, however, is pending to be tested because the participants in Yoshinaga-Itano et al.’s (2010) study were older than the participants in the other three studies. It was also possible that the age-equivalent language performance at age 7 was primarily a matter of chronological maturation.

On the other hand, congenital hearing loss is not limited to specific language populations. The prevalence of bilateral hearing loss is similar across regions worldwide (1/1000 to 3/1000), according to a report by World Health Organization (2009). There is a certain degree of commonality of basic memory processes among people from different language populations. The present study could add a piece of information concerning the universality of the relationship between memory processes and language growth in hearing-impaired children of a non-alphabetic language population.

In order to test the above hypothesis, this study was designed to collect information about span of working memory and outcome of the two types of language skills from a group of Mandarin-speaking children with mild to profound hearing impairment. The children’s language performances were also compared with another group of hearing children. Most importantly, data analysis was conducted with the purpose of examining the influence of the memory span on the relationship between rehabilitation and outcomes of the two types of language skills in the hearing-impaired children.

## Materials and Methods

### Participants

The participants were 39 children with congenital, bilateral hearing loss and 20 children with normal hearing. All of the participants attended kindergarten. The hearing children and 37 of the hearing-impaired children were in regular classes. The hearing children were from three different schools, and those who went to the same school were also classmates. The schools of the hearing-impaired children were different from one another. Two of the hearing-impaired children were from two schools for the hearing impaired. The chronological age of the hearing-impaired children ranged from 5:0 to 6:1 (mean = 5:5), and the number of girls and boys were 18 and 21. Children with multiple disabilities were excluded from this study. All of the hearing-impaired children were wearing hearing aids before and at the time of attending the present study. The age of the hearing participants ranged from 5:11 to 6:5 (mean = 6:2), and the number of girls and boys were 10 and 10. All of them were native speakers of Mandarin, and according to their school teachers, none of the hearing participants was found to have sensory, neurological or psychological disorders. This study was carried out in accordance with the recommendations of IRB Review Guidelines, Chang Gung Medical Foundation Institutional Review Board (IRB No. 103-3749D) with written informed consent from the parents of the participants.

All of the hearing-impaired children have joined a rehabilitation program that adopted an auditory-verbal approach. For each of the children, once the rehabilitation program began, an Ongoing Assessment Form was used to keep track of the child’s development of comprehensive and expressive language. The form was originally designed by Simser (2009) for children who aimed to learn English in auditory-verbal therapy programs. To make the form useful for children who aimed to learn Mandarin, a Chinese version of the form was constructed following the same design principles as the original one. Moreover, a list of 3,467 common words, sampled from two corpora of spoken mandarin (National Languages Committee, 1999; see Tseng, 2013) and a dictionary for children (National Languages Committee, 2000), was included in the form as a checklist to indicate which words the child was able to comprehend or to produce.

When the hearing-impaired child began to be able to comprehend and respond to spoken words, the child’s working memory span was monitored and documented in each session of rehabilitation. The technique used to evaluate the child’s memory span was the same as the “Secret Code” activity described by Garber (2013). That is, the child listened to a list of content words in order to accomplish a mission, and the number of words that the child could remember was taken to determine the child’s memory span. For the participants of this study, the length of the lists began with three words, and the lists were designed in reference to a standard test for hearing children (Hung and Chiu, 1998). If the hearing-impaired child was able to remember the list, a new list was generated by increasing the length of the list by one word. There were two trials for each length of the lists, and the words in the newer lists were not used in previous trials. If the child failed in both trials, the activity stopped. According to the child’s performance in the activity, the child’s memory span was annotated as 3, 4, 5, or 5+ (more than five) words. In order to ensure that the memory spans were tested with materials that the hearing impaired children could easily understand, the words used in the working memory task were selected according to the word checklist of the children’s Ongoing Assessment Forms. Only the words that the child could recognize were used to evaluate the child’s memory span. As a result, the words to be remembered could differ across the hearing-impaired children.

A previous normative study indicated that 6-year-old Taiwanese children could recall 5–6 items in the WISC-III Digit Span sub-test (Chen and Hung, 2004). Therefore, the hearing-impaired children of this study were assigned to the high-span group if the children’s memory span was or more than five words (*n* = 20). Otherwise, the children were assigned to the low-span group (*n* = 19). Results of a Kruskal–Wallis test indicated that the memory span of the high-span group (range = 5.0–6.0, mean = 5.1, 1st quartile = 5.0, 3rd quartile = 5.0) and the memory span of the low-span group (range = 3.0–4.0, mean = 3.9, 1st quartile = 4.0, 3rd quartile = 4.0) differed significantly (*p* < 0.01). On the other hand, the age of the high-span group (range = 5:0–6:1, mean = 5:7,1st quartile = 5:2, 3rd quartile = 5:11) and the age of the low-span group (range = 5:0–6:0, mean = 5:4, 1st quartile = 5:1, 3rd quartile = 5:5) were similar (*p* = 0.06), and the hearing group was older than the high-span group (*p* < 0.01) and the low-span group(*p* < 0.01). The number of children in each level of hearing loss was counted for each group of memory span. As listed in **Table 1**, the levels of hearing loss ranged from mild to profound in both groups of the participants. In addition, information about the length of rehabilitation in months was collected for each of the hearing-impaired participants.

**Table 1:** Number of the hearing-impaired children as a function of levels of memory span and hearing loss.

		Levels of hearing loss				
		Mild	Moderate	Moderate-severe	Severe	Profound
Memory span	High	2	2	4	0	12


	Low	2	6	3	3	5


### Test Material

The revised version of the Language Disorder Assessment for Preschooler (LDAP-R) was administered to the participants of this study. The assessment instrument is the most widely used standardized test to evaluate receptive and expressive language skills of pre-school children in Taiwan (Wang and Lin, 2008). The LDAP was established in Lin and Lin (1994), and it was thoroughly revised in Lin et al. (2008). The LDAP-R consists of four sub-tests, which are the sub-test for quick assessment of speech fluency, the sub-test for receptive language, the sub-test for object naming and the sub-test for expressive language. For the purpose of this study, only the results of the second and the fourth sub-tests were analyzed and reported.

The sub-test of receptive language consists of 37 items. In 23 of the receptive items, each of the participants was presented with a picture of multiple different objects. Then the participants heard a sentence and were asked to point out the object described by the sentence. In 10 of the items, the participants listened to a short story and answered questions about who, where, when and what. In three of the items, the participants listened to a question like “Which is the place for eating, a restaurant or a store,” and gave their answers by making a judgment. In one of the items, the participants were asked to act out a sentence like “Turn your head, and then open your mouth.”

The sub-test of expressive language consists of 46 items. In 24 of the expressive items, the participants were presented with a picture and were asked to describe what happened in the picture as much as they could. In 11 of the items, the participants answered questions of common knowledge. In six of the items, the task was sentence repetition, and in another five of the items, the participants were asked to repeat a description of a picture.

### Procedure

The test was administered individually in a room. The room was kept as quiet as possible while background noise was mainly from an air conditioner. The noise level was at 41 dB(A), measured by a sound level meter (model TES-1350A, TES Electrical Electronic, Corp.). Before the test began, the purpose of this study and the tasks that the participants were going to perform was fully explained to the parents or the caregivers of the participants. The participants consisted of children with hearing impairment and children with normal hearing. Therefore, the test giver also made sure that the participants were comfortable to take the test. By following the manual of the assessment instrument, the sub-tests were administered in a fixed order in which the receptive sub-test was administered before the expressive sub-test for every participant. It took the participants about 20–30 min to finish the test.

### Data Analysis

For each participant, the percentage of items that were corrected answered in each of the two sub-tests was calculated independently, and the results were taken as accuracy. Then the accuracy values from each of the two hearing-impaired groups were compared, respectively, to the accuracy values from the hearing group. Because the majority of the participants were sampled from an atypical developmental population and the group size in this study was relatively small, box-plots were used to present the distribution of the accuracy for each group of the participants. In addition, the Kruskal–Wallis test was used to conduct the statistical testing of difference between the hearing group and the hearing-impaired groups.

In order to examine whether or not the effect of rehabilitation on the measure of language outcome was influenced by the children’s working memory span, a linear mixed-effects model analysis (LMM) was performed for the hearing-impaired group. The LMM analysis has been used in some previous studies to examine possible relationships among multivariate cognitive and linguistic measures from the same participants in different conditions (Nicenboim et al., 2015; Koerner et al., 2016). Therefore, a LMM analysis was conducted in the present study to examine the relationship between memory span and language outcome. The memory span and sub-test type was entered into the model as the fixed effects. As random effects, the model included intercepts for the length of rehabilitation and random slopes of rehabilitation length for the effect of memory span.

All of the statistical analyses were performed by using R Core Team (2015). The R package ‘stats’ was used to conduct the Kruskal–Wallis test. The package nlme (Pinheiro et al., 2016) was used to conduct the LMM analysis.

## Results

The following report of the participants’ performances was organized according to the type of the sub-tests. The results from the test of receptive language were reported before the results from the test of expressive language. Then, whether or not the memory span influenced the relationship between rehabilitation and the two measures of language outcome was reported.

### Receptive Subtest

**FIGURE 1:**
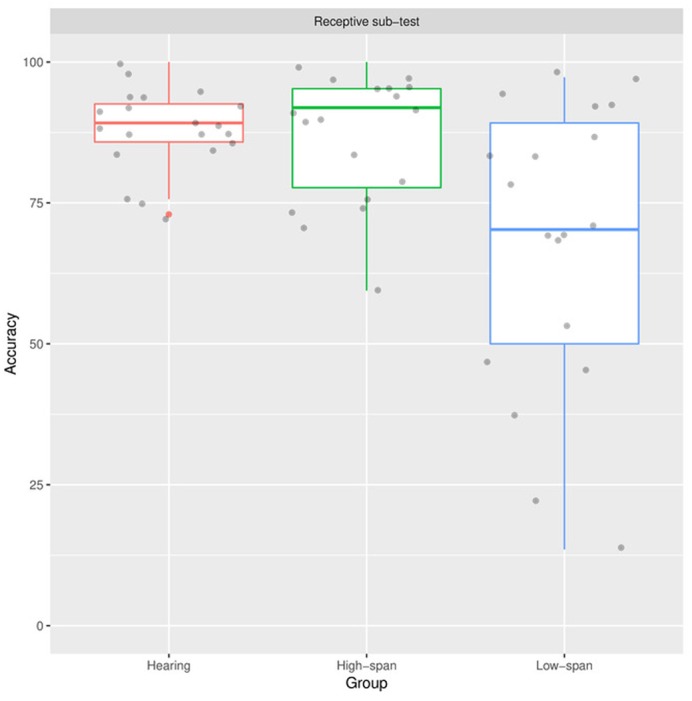
**Distributions of the accuracy (in %) from the Receptive sub-tests for the three groups of participants (Hearing: participants with normal hearing, High-span: participants with hearing loss and high memory span, Low-span: participants with hearing loss and low memory span)**.

As shown in **Figure 1**, the low-span group showed lower accuracy than the high-span group and the hearing group. In most of the participant in the low-span group, the accuracy was lower than 75%. On the contrary, the accuracy was higher than 75% in more than half of the participants from the high-span group and the hearing group.

The result of statistical testing showed that there were significant group differences (χ^2^ = 9.27, *p* < 0.01). Results of planned comparisons confirmed that the difference between the low-span group and the hearing group was significant (*p* = 0.03). On the other hand, the accuracy was similar between the high-span group and the hearing group (*p* = 1.00). The pattern of the results verified the idea that the memory span of the children with hearing loss has an influence on the children’s performances in language comprehension.

### Expressive Subtest

**FIGURE 2:**
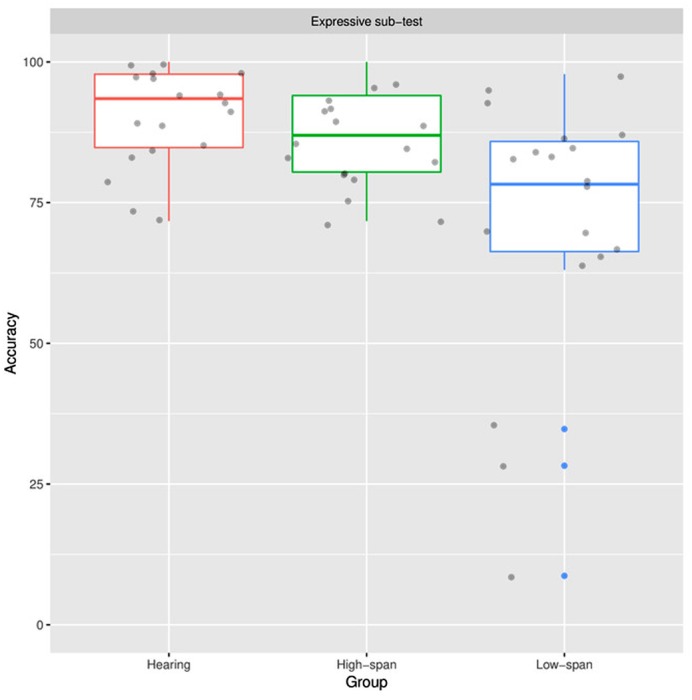
**Distributions of the accuracy (in %) from the Expressive sub-test for the three groups of participants (Hearing: participants with normal hearing, High-span: participants with hearing loss and high memory span, Low-span: participants with hearing loss and low memory span)**.

As shown in **Figure 2**, the pattern of group differences was quite similar to the pattern observed in the results from receptive sub-test. That is, there were significant group differences (χ^2^ = 12.57, *p* < 0.01). Results of planned comparisons showed that the high-span group and the hearing group were not different from each other statistically (*p* = 0.39). In contrast, the group of low memory span showed lower accuracy than the group with normal hearing (*p* < 0.01).

The pattern of the results showed the advantage of the hearing-impaired children with high memory span. The hearing-impaired children could perform as good as the hearing children in language comprehension and production when they have an enough span of temporary memory to process linguistic inputs. In addition, the variation was large in the low-span group while the variation was relatively small in the high-span group. It suggested that the children in the high-span group were less likely to deviate from the typical trajectory of language development than those in the low-span group.

### Influence of Working Memory Span

Linear mixed-effects models, as described above, were constructed by using memory span and sub-test type as the fixed effects and by using rehabilitation length as the random effect. Visual inspection of residual plots showed that deviations from homoscedasticity or normality were not noticeable. To attain the *p*-value of the random effect, Likelihood Ratio Tests of the full model with the effect in question against the model without the effect in question were conducted.

The results of the LMM analysis and the likelihood ratio tests showed the followings: (1) the memory span was a significant predictor of the language outcomes [*F*(1,54) = 6.77, *p* = 0.01]; (2) the sub-test type was not significant [*F*(1,54) = 0.17, *p* = 0.68]; (3) the effect of rehabilitation length was significant (*p* < 0.01). Overall, the results indicated that both the rehabilitation length and the memory span affected the measure of language outcome.

In order to verify whether or not the effect of rehabilitation on the measure of language outcome was influenced by the children’s working memory span, Pearson’s correlations between the rehabilitation length and two measures of language outcome were computed respectively for the high-span and low-span groups. The accuracy in the expressive sub-set positively correlated with the rehabilitation length in the high-span group (*r* = 0.53, *p* = 0.02) but not the low-span group (*r* = 0.03, *p* = 0.90). The correlation between the rehabilitation length and the accuracy in the receptive sub-test was not statistically significant either in the high-span group (*r* = 0.35, *p* = 0.13) or the low-span group (*r* = -0.12, *p* = 0.63).

## Discussion

The results of the present study support the idea that working memory capacity affects outcomes of language learning of Mandarin-speaking children with hearing loss. For the children with hearing loss and high memory span, their performances were almost as good as the hearing children, not only in receptive language but also in expressive language. In contrast, the children with hearing loss and low memory span showed lower scores than the hearing children in the receptive and expressive language tests.

The above finding suggests that the outcome of language learning of the hearing-impaired children depend partly on the children’s working memory capacity. The children with hearing loss could efficiently assess task-relevant information with the help of a large span of working memory. As a result, they are at an advantage when taking the language tests than those who have a small span of working memory. It is consistent with the literature which shows that there is a positive correlation between memory function and language development among ordinary children (Kidd, 2013). In other words, the mechanism underlying the relationship between working memory and language acquisition could also account for the process of language learning among hearing-impaired children.

However, for children with hearing loss, the outcome of language learning could be more sensitive to the children’s working memory capacity. Some previous studies have shown that children with congenital hearing loss have lower scores on the tests of working memory than do hearing children (Pisoni and Cleary, 2003; Pisoni et al., 2011). When the hearing-impaired children were asked to process multiple types of information at the same time, working memory is likely to be overloaded with the information. In other words, children with hearing loss may not have enough memory resource to complete a task that children with normal hearing can easily coped with. In the case of taking a language test, children with hearing loss could be more likely to be overwhelmed by the amount and processing of phonological, semantic and syntactic information. Furthermore, working memory demand could be different between the test of receptive language and the test of expressive language. Test takers have to work on two sources of linguistic information to perform the test of expressive language. One is provided by a test giver. The other is generated by the test takers themselves. Logically, working memory demand was relatively high in the test of expressive language.

Capacity of working memory could be enhanced by systematic training and learning (Dunning et al., 2013; Harrison et al., 2013). According to the research by Ingvalson et al. (2014), children with hearing loss can take advantage of working memory training to overcome the limit of processing capacity. Take the performance in the test of expressive language as an example, Ingvalson et al. found that the children at age 4:7–6:7 showed significant improvement at expressive language scores after receiving working-memory training. Similarly, the present study found that the rehabilitation length positively correlates with the accuracy in the expressive language test only among the children with high memory span. Nevertheless, how much the children’s working memory span could be explained by rehabilitation was not conclusive. According to Ericsson and Kintsch’s (1995) model of working memory, extensive knowledge acquired from experience in a particular domain can enhance the efficiency of memory storage and retrieval. If capacity of working memory could be enhanced by rehabilitation, there could be a bi-directional relationship between working memory and rehabilitation. More research is needed to test this idea.

This study also has implication for the design of rehabilitation plans for children with hearing impairment. Recently, a question of whether or not measures of working memory should be incorporated into hearing aid decision was raised (Souza, 2012). Working memory capacity and language ability are crucial to the academic performance of school children (Gathercole et al., 2006). If the hearing-impaired children and their caregivers prefer to know the extent to which the children’s memory and language ability are ready for the children to go school, monitoring and training of working memory should probably be a necessary part of rehabilitation plans for the children. Moreover, when lists of words are used as test items to measure the working memory capacity, it is important to ensure that the test words are familiar to the children. Only if the familiarity of the words is high, which was controlled in the present study and some standard tests for hearing children (Hung and Chiu, 1998; Chien et al., 2014), the possibility that the children’s working memory performances are confounded with the children’s difficulties in language comprehension could be largely reduced.

In summary, the results of the present study verify the hypothesis that language acquisition of Mandarin-speaking children with hearing loss is under the influence of working memory capacity. Not only acquisition of receptive language but also acquisition of expressive language relates to the specific memory ability. Sufficient working memory capacity is one of the factors that could support the children to acquire age-equivalent language skills. However, whether or not working memory capacity acts as a mediator needs to be verified in future research.

## Author Contributions

Both authors contributed equally to this work: ML shaped the design and conception of the study, performed data analysis and wrote the first draft; P-HC designed the study, carried out data acquisition and edited several sections.

## Conflict of Interest Statement

The authors declare that the research was conducted in the absence of any commercial or financial relationships that could be construed as a potential conflict of interest.
